# RANKL and OPG Polymorphisms Are Associated with Aromatase Inhibitor-Related Musculoskeletal Adverse Events in Chinese Han Breast Cancer Patients

**DOI:** 10.1371/journal.pone.0133964

**Published:** 2015-07-28

**Authors:** Jingxuan Wang, Kangping Lu, Ying Song, Shu Zhao, Wenjie Ma, Qijia Xuan, Dabei Tang, Hong Zhao, Lei Liu, Qingyuan Zhang

**Affiliations:** Department of Medical Oncology, The Third Hospital of Harbin Medical University, Harbin, China; Inserm U606 and University Paris Diderot, FRANCE

## Abstract

**Background:**

Breast cancer patients treated with aromatase inhibitors (AIs) may experience musculoskeletal adverse events (MS-AEs). Several studies have confirmed that the RANKL/RANK/OPG signaling pathway plays a dominant role in bone health. Therefore, this study aimed to analyze the relationship between the serum levels of RANKL, OPG and their SNPs (single nucleotide polymorphisms) with AI-related MS-AEs.

**Methodology and Principal Findings:**

Patients with early stage, hormone-sensitive breast cancer who were receiving AI therapy were enrolled. We included 208 cases with AI-related MS-AEs and 212 without (controls). The levels of estradiol, bone-turnover markers, multiple inflammatory cytokines, RANKL,OPG and lumbar spine BMD were measured, and questionnaires were completed. We analyzed 29 SNPs of RANKL, RANK and OPG using Sequenom MassARRAY assays and PCR-based TaqMan assays. The levels of bone-turnover markers and RANKL and the ratio of RANKL/OPG were higher in patients with AI-related MS-AEs than controls (all p < 0.05). A genetic assay showed that the RANKL SNP rs7984870 and OPG SNP rs2073618 were associated with AI-related MS-AEs. In patients with AI-related MS-AEs, rs7984870 CC and rs2073618 CC were risk genotypes. Carriers of the rs7984870 CC genotype were more likely to have a higher RANKL level and RANKL/OPG ratio than carriers of the GG genotype, and carriers of the rs2073618 CC genotype were more likely to have a lower OPG level and a higher RANKL/OPG ratio than carriers of the GG genotype (all p < 0.05). Moreover, risk genotypes were associated with higher levels of serum CTX and PINP and a lower lumbar spine BMD (all p < 0.05).

**Conclusions and Significance:**

In conclusion, the RANKL and OPG risk genotypes synergize to negatively impact bone health and predispose breast cancer patients to AI-related MS-AEs.

## Introduction

Tamoxifen has been the mainstay of endocrine therapy for more than 30 years. However, recent studies have indicated that aromatase inhibitors (AIs) are superior to the antiestrogen tamoxifen for improving the rates of disease-free survival and possibly overall survival in postmenopausal women with hormone receptor-positive breast cancer [1–3]. AIs profoundly reduce circulating estrogen levels in postmenopausal women by an additional 80–90% compared with tamoxifen. However, blocking estrogen synthesis is associated with a modest increase in deleterious effects on the musculoskeletal system, such as arthralgia, osteoporosis, and bone fractures [4], which are referred to as AI-related musculoskeletal adverse events (MS-AEs) [5,6]. MS-AEs are of particular importance among adverse events in early stage breast cancer patients. Because these patients may be cured and have a life expectancy in the decades, it is crucial to avoid the potential fractures and deformity associated with MS-AEs. Consequently, an understanding of the risks of disease progression and the therapeutic options available for the maintenance and restoration of their bone health is imperative for individual patients with early stage breast cancer.

Although no agents have received broad international regulatory approval for the prevention of AI-related MS-AEs, several interventions have demonstrated activity against aromatase inhibitor-induced bone loss and musculoskeletal symptoms, including denosumab, zoledronic acid and acupuncture [5–9]. Denosumab, a fully human monoclonal antibody against RANKL, has been approved for the treatment of osteoporosis in postmenopausal women in the United States (in the event that other therapies have failed or are contraindicated) and Europe. Subgroup analyses of a phase 3 study showed that twice-yearly administration of denosumab consistently increased BMD (bone mineral density) versus placebo at 12 and 24 months for nonmetastatic breast cancer patients receiving adjuvant aromatase inhibitor therapy [10].

Bones are continuously repaired in adults by well-organized cycles of bone resorption and formation, so-called “bone remodeling”. The RANKL/RANK/OPG signaling pathway plays an important role in the regulation of bone remodeling, osteoclast differentiation and osteolysis [11]. RANKL, a type II membrane protein of the tumor necrosis factor family (*TNFSF11*), is expressed on osteoblasts, stromal cells, activated T cells, B cells and megakaryocytes. Bone loss is mediated by osteoclasts, whose formation, function, and survival all depend on RANKL activity. Binding of RANK to RANKL on preosteoclasts and mature osteoclasts can promote the formation, activation, and survival of multinucleated osteoclasts during normal bone remodeling and a variety of pathologic conditions [12,13]. OPG, a decoy receptor for RANKL, protects bone from excessive resorption via binding to RANKL and thereby preventing it from binding to RANK [12,13]. The genes encoding these proteins are well known as candidate genes that influence the maintenance of bone health [14,15].

In our previous study, we reported two ESR1 SNPs, rs2234693 and rs9340799 were associated with AIs-related MS-AEs. Based on these findings, we selected 29 SNPs of RANKL, RANK and OPG in the present study to identify whether they were associated with MS-AEs in women receiving adjuvant AI therapy for early stage breast cancer. Furthermore, we performed studies to elucidate the potential functional basis for these associations.

## Materials and Methods

### Ethics statement

We obtained approval from the ethical board of the Third Hospital of Harbin Medical University, and all of the volunteers provided written informed consent prior to conducting this study.

### Study participants

From August 2007 to March 2012, we recruited postmenopausal Chinese Han women with completely resected, stage I to III breast cancer in the Third Affiliated hospital of Harbin Medical University. Pathologically, all of the patients had estrogen receptor (ER)-positive and/or progesterone receptor (PR)-positive breast cancer. To be considered for enrollment, patients needed to be currently taking letrozole (2.5 mg/day) or anastrozole (1 mg/day) adjuvant therapy, and this regimen must have been taken for at least 1 year prior to the study. All indicated surgeries, radiation therapies, and chemotherapies for breast cancer treatment were completed before enrollment.

### Patient definition for musculoskeletal adverse events

Because the patients received AI therapy, they had at least one of the following six symptoms of MS-AEs: joint pain, muscle pain, bone pain, arthritis, diminished joint function, or other musculoskeletal problems. Cases were required to have at least grade 3 toxicity according to the National Cancer Institute’s (NCI) Common Terminology Criteria for Adverse Events v3. Patients who had either of the following were excluded from the study: (1) any MS-AEs before they received AI therapy or (2) inflammatory, metabolic, or neuropathic arthropathies or surgery of an afflicted extremity during the preceding 6 months and were currently taking steroids (oral or injected) or narcotics.

Controls were breast cancer patients who were completely free of any MS-AEs after AI therapy and had not taken steroids (oral or injected) or narcotics for at least 6 months.

Patients and controls were matched for demographics, clinical characteristics and prior therapy. At the time of evaluation, rheumatologists reported the symptoms’ severity and impact on patient function, as well as the apparent association between symptoms and AI therapy (related or not related).

### Measurements of BMD

The BMD of the lumbar spine (L1-4) was measured by dual energy X-ray absorptiometry (Lunar prodigy, GE Healthcare, United Kingdom). BMD was reported as an absolute value (g/cm2) and as a T score, which represents the number of standard deviations from a young, sex- and ethnic group-specific reference mean. According to the World Health Organization’s (WHO) definitions, T scores were used as the basis for diagnosis as follows: normal bone mineral density, T score greater than -1; osteopenia, T score less than or equal to -1 but greater than -2.5; and osteoporosis, T score less than or equal to -2.5.

### Specimen collection and DNA extraction

Whole blood was obtained for genotyping and measuring inflammatory markers. All samples were examined blindly by laboratory personnel. Serum was obtained by centrifugation at 1000 × g for 5 min at room temperature. DNA samples were extracted from peripheral blood samples of patients and controls using the AxyPrep Blood Genomic DNA Miniprep Kit (Axygen Biotechnology, USA). Serum samples were stored at -80°C until analysis. Each DNA sample was stored at -20°C until analysis.

### Assessment of serum estradiol, bone turnover markers, inflammatory markers and OPG/RANK/RANKL

Serum estradiol (E2) was measured using an electrochemiluminescence immunoassay (ECLIA; Roche Diagnostics GmbH). Two bone turnover markers, carboxy terminal telopeptide (CTX) and procollagen type I N-terminal propeptide (PINP), were measured in serum samples via ECLIA (Roche diagnostics GmbH).

Levels of inflammatory markers, including interferon gamma (IFNγ), tumor necrosis factor alpha (TNFα), interleukin-1 (IL1), interleukin-4 (IL4), interleukin-6 (IL6), interleukin-10 (IL10), interleukin-12 (IL12), interleukin-17 (IL17) and interleukin-23 (IL23), were measured. The concentrations of the serum inflammatory markers OPG, RANK and RANKL in supernatants were assessed using an enzyme-linked immunosorbent assay (ELISA) kit according to the manufacturer’s instructions (R&D Systems, Minneapolis, MN).

### SNP selection

Known SNPs of OPG, RANK and RANKL were selected using “Tagger” (http://www.broadinstitute.org/mpg/tagger/server.html) and data from the International HapMap Project (http://snp.cshl.org/). A total of 27 SNPs of the three genes of interest (7 for OPG, 16 for RANK, 4 for RANKL) were selected based on the following criteria: (1) their status was validated in a Han Chinese population in Beijing; (2) their tag SNPs were in linkage disequilibrium with all SNPs in the HapMap dataset with a minor allele frequency ≥ 5%; and (3) they were identified after running the Tagger program in an aggressive tagging mode using 2- and 3-marker haplotypes with r² and LOD thresholds set at 0.8 and 3.0 respectively.

The SNPs rs2277438 in the RANKL gene and rs2073618 in the OPG gene were also selected because they are associated with bone health [16,17]. rs2073618 is a missense mutation of the OPG gene, while other 28 SNPs are located in the intron region of each gene.

### Genotyping

Genotyping was performed by the Sequenom MassARRAY technology platform using iPLEX GOLD chemistry (Sequenom, San Diego, CA). Briefly, specific assays were designed using the MassARRAY AssayDesign software package with filtering of proximal SNPs and checking of specificity for PCR amplification and the subsequent primer extension reaction. SpectroTyper 4.0 was used to call genotypes automatically, followed by manual review.

As a secondary platform, PCR-based TaqMan assays (Applied Biosystems, Foster City, CA) were performed according to the manufacturer’s instructions, and the results were analyzed on the ABI Prism 7500 using Sequence Detection Software (Applied Biosystems Co. Ltd., USA). To confirm the accuracy of the genotyping results, 10% of the samples of each SNP were randomly selected to be tested twice by different lab personnel; the reproducibility was 100%.

### Statistical analysis

The non-parametric Mann-Whitney U-test was used to compare case and control values for categorical data, and unpaired two-tailed t-tests were used for continuous data. The strength of pair-wise linkage disequilibrium (LD) (r2) between two genetic polymorphisms was assessed using Haploview 4.1. Logistic regression analyses were used to estimate the odds ratios (OR), 95% confidence intervals (CI) and corresponding p values of alleles and genotypes, with OR values above 1.0 indicating an increased risk. Data were analyzed using SPSS 19.0. A p value < 0.05 was considered to indicate statistical significance. For evaluating the Sequenom MassARRAY assay data of the 29 SNPs of RANK, RANKL and OPG, p values < 0.05 were defined as nominally significant, bonferroni correction for multiple testing was applied for the total number of SNPs in this study when assessing relationship between Minor Allele Frequency and individual SNPs (the corrected level of significance was p = 0.05/29 = 0.0017).

## Results

### Patient characteristics

We selected 208 patient cases who were referred for rheumatological evaluation because of the following six MS-AEs: joint pain, muscle pain, bone pain, arthritis, diminished joint function or worsened functional status during AI therapy. We then selected 212 matched controls who did not experience increased pain or worsened functional status during AI therapy. The baseline characteristics of patients in the AI-related MS-AEs group and control group are presented in Table 1. Serum estradiol, CTX, PINP, OPG, RANKL concentration and BMD prior to aromatase inhibitors treatments in the AI-related MS-AEs group and control group are presented in S1 Table.

**Table 1:** Subject characteristics of the patients in AIs-related MS-AEs group and controls.

Characteristic	Cases (n = 208)		Controls (n = 212)		
	n	%	n	%	p value
**Age (years)**					0.769
≥58	102	49.0	107	50.5	
<58	106	51.0	105	49.5	
Mean ± SD	57.3 ±15.7		58.1 ±14.9		0.091
**Treatment arm**					0.783
Anastrozole	68	32.7	72	34.0	
Letrozole	140	67.3	140	66.0	
Median treatment time (months)	24.9± 7.6		25.3± 8.2		0.145
**ER/PR status**					0.653
ER+/PR+	133	63.9	140	66.0	
ER+/PR–	58	27.9	60	28.3	
ER–/PR+	17	8.2	12	5.7	
**Her-2 status**					0.978
Negative	142	68.3	145	68.4	
Positive	66	31.7	67	31.6	
**Histological grade**					0.846
I	30	14.4	32	15.1	
II	146	70.2	147	69.3	
III	32	15.4	33	15.6	
**Years since menopause**					0.698
≥10	65	31.2	70	33.0	
<10	143	68.8	142	67.0	
**Median BMI (kgm** ^**-2**^ **, range)**					0.773
≥25	105	50.5	110	51.9	
<25	103	49.5	102	48.1	
**Prior chemotherapy**					0.580
No	66	31.7	62	29.2	
Yes	142	68.3	150	70.8	
**Prior taxane treatment**					0.145
No	120	65.5	137	68.2	
Yes	88	34.5	75	31.8	
**Prior tamoxifen treatment**					0.591
No	172	82.7	171	80.7	
Yes	36	17.3	41	19.3	
**Prior radiation therapy**					0.894
No	67	32.2	67	31.6	
Yes	141	67.8	145	68.4	
**Smoking**					0.781
No	190	91.3	192	90.6	
Yes	18	8.7	20	9.4	

Note: SD = standard deviation; BMI = body mass index.

### Serum concentrations of estradiol, bone turnover markers, and multiple inflammatory markers

Serum concentrations of estradiol and bone turnover markers from patients in the AI-related MS-AEs group were compared with those of the controls (Table 2). As expected, both groups had an average estradiol level in the postmenopausal range. Mean estradiol levels were significantly lower in the AI-related MS-AEs group compared with the controls (8.98 ± 5.92 pg/ml vs. 11.23 ± 6.22 pg/ml; p = 3.16E-7).

Furthermore, we observed significant differences in the concentrations of CTX (231.1 ± 113.7 pg/ml vs. 219.8 ± 98.2 pg/ml; p = 8.46E-6) and PINP (357.1 ± 134.3 pg/ml vs. 332.6 ± 118.9 pg/ml; p = 3.64E-7) between cases and controls.

**Table 2:** Serum concentrations of serum estradiol, bone turnover markers and inflammatory markers.

	Cases	Controls	
	(mean ± SD)	(mean ± SD)	p value
**E2(pg/ml)**	8.98 ± 5.92	11.23 ± 6.22	**3.16E-7**
**CTX(pg/ml)**	231.1 ± 113.7	219.8 ±98.2	**8.46E-6**
**PINP(pg/ml)**	357.1± 134.3	332.6 ± 118.9	**3.64E-7**
**IFNγ(pg/ml)**	318.7 ± 157.3	308.5 ± 155.1	0.076
**TNFα(pg/ml)**	19.15 ± 4.17	18. 71 ± 3.99	0.111
**IL1(pg/ml)**	77.32 ± 35.58	75.01 ± 23.60	0.332
**IL4(pg/ml)**	24.02 ± 5.90	24.94 ± 5.86	0.092
**IL6(pg/ml)**	5.95 ± 1.36	6.21 ± 1.94	0.115
**IL10(pg/ml)**	24.65 ± 3.99	24.32 ± 2.35	0.081
**IL12 (pg/ml)**	2.46 ± 0.77	2.56 ± 0.85	0.255
**IL17(pg/ml)**	13.55 ± 5.42	12.67 ± 4.93	0.067
**IL23(pg/ml)**	894.1 ± 341.0	908.3 ± 386.2	0.075

Note: SD = standard deviation; E2 = estradiol; CTX: carboxy terminal telopeptide;PINP: procollagen type I N-terminal propeptide.

Among the cytokines with the greatest importance in inflammatory responses, pain and arthritis, we selected nine cytokines for further study (IFNγ, TNFα, IL1, IL4, IL6, IL10, IL12, IL17 and IL23). No significant differences were observed in any of these inflammatory factors between cases and controls.

**Fig 1:**
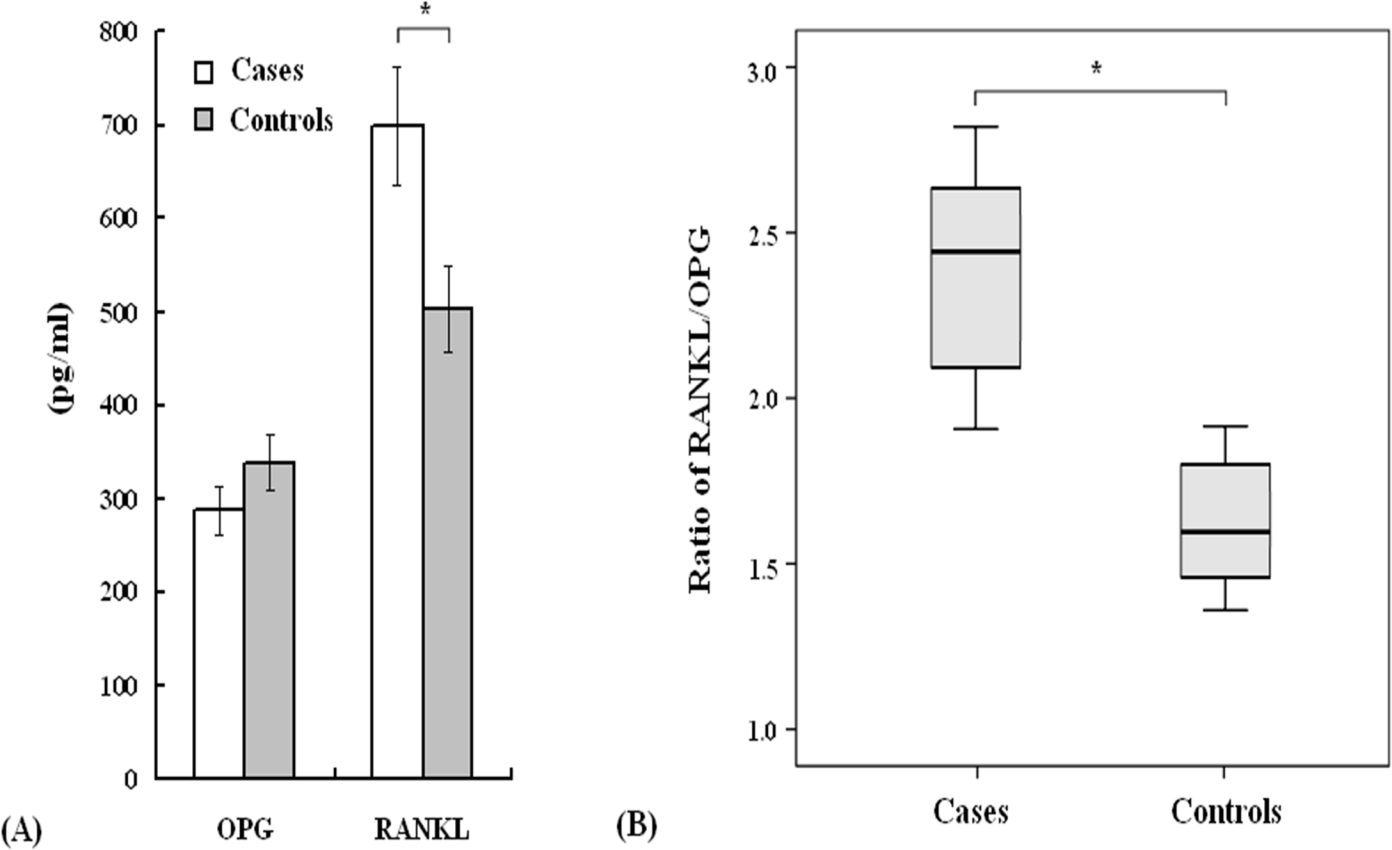
RANKL and OPG expression in 420 breast cancer patients. **A. Serum concentrations of RANKL and OPG. B.** The ratio of RANKL/OPG. Cases: n = 208; Controls: n = 212. *p < 0.05.

Next, the serum concentrations of OPG and RANKL were analyzed in matched pairs of cases and controls (Fig 1). The serum OPG levels in the AI-related MS-AEs group were reduced compared with controls, although this difference was not significant. However, for breast cancer patients with AI-related MS-AEs, the level of RANKL (698.0 ± 62.6 pg/ml) and the ratio of RANKL/OPG (2.43 ± 0.22) were higher than the controls (RANKL: 502.1 ± 45.2 pg/ml, RANKL/OPG: 1.48 ± 0.14, p < 0.05).

### Frequencies of genotypes and alleles

A total of 29 SNPs of the three genes were selected (8 for OPG, 16 for RANK, 5 for RANKL). The LD patterns of these three genes are shown in S1 Fig None of the genotyped SNPs had a minor allele frequency less than 5%. To ensure the quality control of genotyping results, samples that failed to be genotyped in the first round were genotyped a second time. As shown in Table 3, a lower prevalence of the rs7984870 minor allele was observed in breast cancer patients with AI-related MS-AEs (39.4%) than the controls (60.6%), and this association was significant (p = 3.07E-5, OR = 0.560, 95% CI: 0.425–0.736). The rs2073618 allele was associated with AI-related MS-AEs in this study (p = 6.09E-4, OR = 1.623, 95% CI: 1.233–2.141). However, the other 27 SNPs of RANK, RANKL and OPG showed no significant differences between the case and control groups (p > 0.05).

**Table 3:** Association between risk of AI-related musculoskeletal adverse events and 29 SNPs of RANK, RANKL and OPG.

			Minor Allele Frequency						
dbSNP	Gene	Chr	Position	Location	cases	controls	OR	95%CI	P value
rs12457042	RANK	18	58144433	intron	0.151	0.146	1.042	0.713–1.524	0.847
rs4941129	RANK	18	58151457	intron	0.409	0.413	0.983	0.747–1.294	0.944
rs7237982	RANK	18	58155233	Intron	0.065	0.061	1.062	0.609–1.853	0.888
rs7239261	RANK	18	58156026	Intron	0.216	0.203	1.085	0.778–1.513	0.672
rs12969154*	RANK	18	58157123	Intron	0.324	0.330	0.971	0.727–1.296	0.883
rs4369774	RANK	18	58161428	Intron	0.315	0.311	1.017	0.760–1.361	0.941
rs12956925	RANK	18	58164620	Intron	0.075	0.083	0.895	0.541–1.481	0.702
rs11877530	RANK	18	58166915	Intron	0.267	0.264	1.014	0.746–1.377	0.938
rs11664594	RANK	18	58169186	Intron	0.495	0.486	1.038	0.792–1.361	0.836
rs12455775*	RANK	18	58170830	Intron	0.261	0.250	1.059	0.776–1.444	0.752
rs6567270	RANK	18	58177985	Intron	0.257	0.267	0.953	0.701–1.296	0.814
rs7239667	RANK	18	58180218	Intron	0.452	0.425	1.118	0.851–1.468	0.445
rs4303637^	RANK	18	58182743	Intron	0.363	0.398	0.862	0.652–1.139	0.319
rs4941131	RANK	18	58191141	Intron	0.313	0.318	0.973	0.727–1.302	0.882
rs6567276	RANK	18	58195648	Intron	0.315	0.297	1.087	0.811–1.458	0.601
rs9646629	RANK	18	58202179	Intron	0.377	0.394	0.933	0.706–1.232	0.671
rs7984870	RANKL	13	42044482	Intron	0.394	0.606	0.560	0.425–0.736	**3.07E-5** ^**#**^
rs9594782	RANKL	13	42049186	intron	0.120	0.116	1.046	0.687–1.590	0.835
rs9566990	RANKL	13	42062710	intron	0.231	0.236	0.972	0.706–1.338	0.871
rs9533166	RANKL	13	42075169	intron	0.192	0.167	1.184	0.832–1.684	0.370
rs2277438	RANKL	13	42053168	intron	0.255	0.222	1.200	0.873–1.650	0.292
rs3102724	OPG	8	120015988	intron	0.380	0.384	0.981	0.742–1.295	0.943
rs1485286	OPG	8	120019849	intron	0.423	0.448	0.903	0.687–1.187	0.487
rs1485288	OPG	8	120019969	intron	0.118	0.097	1.247	0.804–1.934	0.323
rs3134058*	OPG	8	120023289	intron	0.454	0.448	1.024	0.780–1.345	0.890
rs11573856	OPG	8	120024176	intron	0.151	0.163	0.918	0.633–1.332	0.705
rs3102728	OPG	8	120025486	intron	0.125	0.118	1.069	0.706–1.617	0.833
rs3134068	OPG	8	120031840	intron	0.111	0.099	1.131	0.727–1.759	0.652
rs2073618	OPG	8	120033233	missense	0.478	0.361	1.623	1.233–2.141	**6.09E-4** ^**#**^

*Cases = 207, missing = 1

^controls = 211, missing = 1

^#^ Significant after Bonferroni correction

We attempted to determine whether any of these SNPs may be functional using Sequenom MassARRAY assays. We then tested and verified these results using PCR-based TaqMan assays. Similar to the results of the MassARRAY assays, rs7984870 of RANKL and rs2073618 of OPG were associated with AI-related MS-AEs. As shown in Table 4, for the rs7984870 polymorphism, we observed a higher prevalence of the CC genotype and C allele (p = 2.19E-4, OR = 1.801, 95% CI: 1.306–2.483; and p = 3.07E-5, OR = 1.787, 95% CI: 1.359–2.351, respectively) in the AI-related MS-AEs group than the control group. Furthermore, the C allele of rs2073618 had a higher prevalence among breast cancer patients with AI-related MS-AEs (p = 6.09E-4, OR = 1.623, 95% CI: 1.233–2.141), and genotype analysis also indicated a significant association between this SNP and the risk of AI-related MS-AEs (CC: p = 7.95E-4, OR = 2.931, 95% CI: 1.624–5.288).

**Table 4:** Genotypes and Allele frequencies of rs7984870 of RANKL and rs2073618 of OPG which had potentially statistical significance between cases in AI-related musculoskeletal adverse events group and control.

Reference SNP ID	Genotype	Frequency no (%)				
		Cases	Controls	P value	OR	95%CI
		(n = 208)	(n = 212)			
rs7984870	GG	32(15.4)	59 (27.8)	**0.002**	1.0	
	CG	100(48.1)	110 (51.9)	0.760	1.676	1.008–2.787
	CC	76 (36.5)	43 (20.3)	**2.19E-4**	3.259	1.843–5.763
G		164(39.4)	228 (53.8)	**3.07E-5**	1.0	
C		252(60.6)	196(46.2)		1.787	1.359–2.351
rs2073618	GG	59(28.4)	83(39.2)	**0.023**	1.0	
	GC	99(47.6)	105(49.5)	0.697	1.326	0.861–2.043
	CC	50(24.0)	24 (11.3)	**7.95 E-4**	2.931	1.624–5.288
G		217(52.2)	271(63.9)	**6.09 E-4**	1.0	
C		199(47.8)	153 (36.1)		1.623	1.233–2.141
rs7984870+ rs2073618						
No risk genotype*		137 (65.9)	180 (84.9)	**5.77 E-6**	1.0	
With risk genotype		71 (34.1)	32 (15.1)		2.915	1.817–4.677

*rs7984870 haplotype was CC or rs2073618 haplotype was CC

Moreover, the haplotypes CC of rs7984870 and CC of rs2073618 were risk genotypes; women with either risk genotype had a significantly higher risk of AI-related MS-AEs than patients without these genotypes (p = 5.77E-6, OR = 2.915, 95% CI: 1.817–4.677).

### Correlation of risk genotypes with estradiol, bone turnover markers and BMD

Table 5 shows differences in the levels of estradiol, CTX, PINP and BMD in relation to the risk genotypes in patients with AI-related MS-AEs.

**Table 5:** Differences of estradiol, CTX, PINP and BMD with risk genotype or no risk genotype in AI-related musculoskeletal adverse events group.

Genotype	Genotype		P value
	Risk	No risk	
	rs7984870 CC	rs7984870 GG	
	(n = 76)	(n = 32)	
E2 (pg/ml)	8.72 ± 4.96	9.11 ± 5.02	0.409
CTX (pg/ml)	242.1 ± 98.1	229.7 ± 86.8	**1.11E-6**
PINP (pg/ml)	371.6± 139.9	336.3 ± 123.1	**1.50E-7**
Lumbar spine BMD (g/cm2)	0.806 ± 0.186	0.975 ± 0.206	0.075
WHO-criteria	n (%)	n (%)	
Lumbar spine BMD T-score			
<−2.5 SD (Osteoporosis)	23(30.2)	3(9.4)	**0.026**
−2.5 SD–−1.0 SD (Osteopenia)	32(42.1)	6(18.8)	**0.027**
−>1.0 SD (Normal)	21(27.2)	23(71.8)	**3.15E -5**
	rs2073618 CC	rs2073618 GG	
	(n = 50)	(n = 59)	
E2 (pg/ml)	8.59 ± 5.06	9.19 ± 5.77	0.181
CTX (pg/ml)	252.6 ± 99.5	223.9 ±84.3	**3.68E-9**
PINP (pg/ml)	369.1± 127.4	325.0 ± 120.2	**5.09E-10**
Lumbar spine BMD (g/cm2)	0.801 ± 0.218	0.987 ± 0.257	**0.049**
WHO-criteria	n (%)	n (%)	
Lumbar spine BMD T-score			
<−2.5 SD (Osteoporosis)	18(36.0)	10(16.9)	**0.029**
−2.5 SD–−1.0 SD (Osteopenia)	20(40.0)	12(20.3)	**0.034**
−>1.0 SD (Normal)	12(24.0)	37(62.7)	**9.20E-5**

Note: E2 = estradiol; BMD = bone mineral density.

No significant differences were observed in serum estradiol levels based on the risk or no risk genotype. However, we observed a significant correlation between the expression of bone turnover markers and risk genotypes. Women with risk genotypes had significantly increased levels of serum CTX (rs7984870: 242.1 ± 98.1 pg/ml vs. 229.7 ± 86.8 pg/ml, p = 1.11E-6; rs2073618: 252.6 ± 99.5 pg/ml vs. 223.9 ± 84.3 pg/ml, p = 3.68E-9) and PINP (rs7984870: 371.6 ± 139.9 pg/ml vs. 336.3 ± 123.1 pg/ml, p = 1.50E-7; rs2073618: 369.1 ± 127.4 pg/ml vs. 325.0 ± 120.2 pg/ml, p = 5.09E-10) compared with women with no risk genotypes.

Compared with no risk genotypes, the rs2073618 CC risk genotype (0.987 ± 0.257 g/cm^2^ vs. 0.801 ± 0.218 g/cm^2^, p = 0.049) but not the rs7984870 CC risk genotype (0.975 ± 0.206 g/cm^2^ vs. 0.806 ± 0.186 g/cm^2^, p = 0.075) showed a significant reduction in BMD at the lumbar spine. However, women with either risk genotype of AI-related MS-AEs had a higher prevalence of osteoporosis (rs7984870: 30.2% vs. 9.4%, p = 0.026; rs2073618: 36.0% vs. 16.9%, p = 0.029) and osteopenia (rs7984870: 42.1% vs. 18.8%, p = 0.027; rs2073618: 40.0% vs. 20.3%, p = 0.034) than women with no risk genotypes.

As it showed in Table 6, we didn’t observe any correlation between the expression of bone turnover markers and risk genotypes in the controls.

**Table 6:** Differences of estradiol, CTX, PINP and BMD with risk genotype or no risk genotype in the control.

Genotype	Genotype		P value
	Risk	No risk	
	rs7984870 CC	rs7984870 GG	
	(n = 43)	(n = 59)	
E2 (pg/ml)	11.16 ± 5.98	11.30 ± 6.10	0.777
CTX (pg/ml)	222.2 ± 89.2	218.9 ± 88.6	0.051
PINP (pg/ml)	334.1± 125.8	331.0 ± 121.0	0.168
Lumbar spine BMD (g/cm2)	0.975± 0.207	1.002 ± 0.225	0.772
WHO-criteria	n (%)	n (%)	
Lumbar spine BMD T-score			
<−2.5 SD (Osteoporosis)	5(11.6)	5(8.5)	0.597
−2.5 SD–−1.0 SD (Osteopenia)	16(37.2)	21(35.6)	0.867
−>1.0 SD (Normal)	22(51.2)	33(56.0)	0.633
	rs2073618 CC	rs2073618 GG	
	(n = 24)	(n = 83)	
E2 (pg/ml)	11.21 ± 5.67	11.28 ± 5.89	0.900
CTX (pg/ml)	223.9 ± 95.9	219.5 ±88.9	0.053
PINP (pg/ml)	335.2± 123.8	332.1 ± 118.5	0.230
Lumbar spine BMD (g/cm2)	0.968 ± 0.211	0.999 ± 0.245	0.775
WHO-criteria	n (%)	n (%)	
Lumbar spine BMD T-score			
<−2.5 SD (Osteoporosis)	3(12.5)	7(8.4)	0.545
−2.5 SD–−1.0 SD (Osteopenia)	9(37.5)	30(36.1)	0.903
−>1.0 SD (Normal)	12(50.0)	46(55.4)	0.639

Note: E2 = estradiol; BMD = bone mineral density.

### The rs7984870 SNP may affect RANKL expression and RANKL/OPG ratio

**Fig 2:**
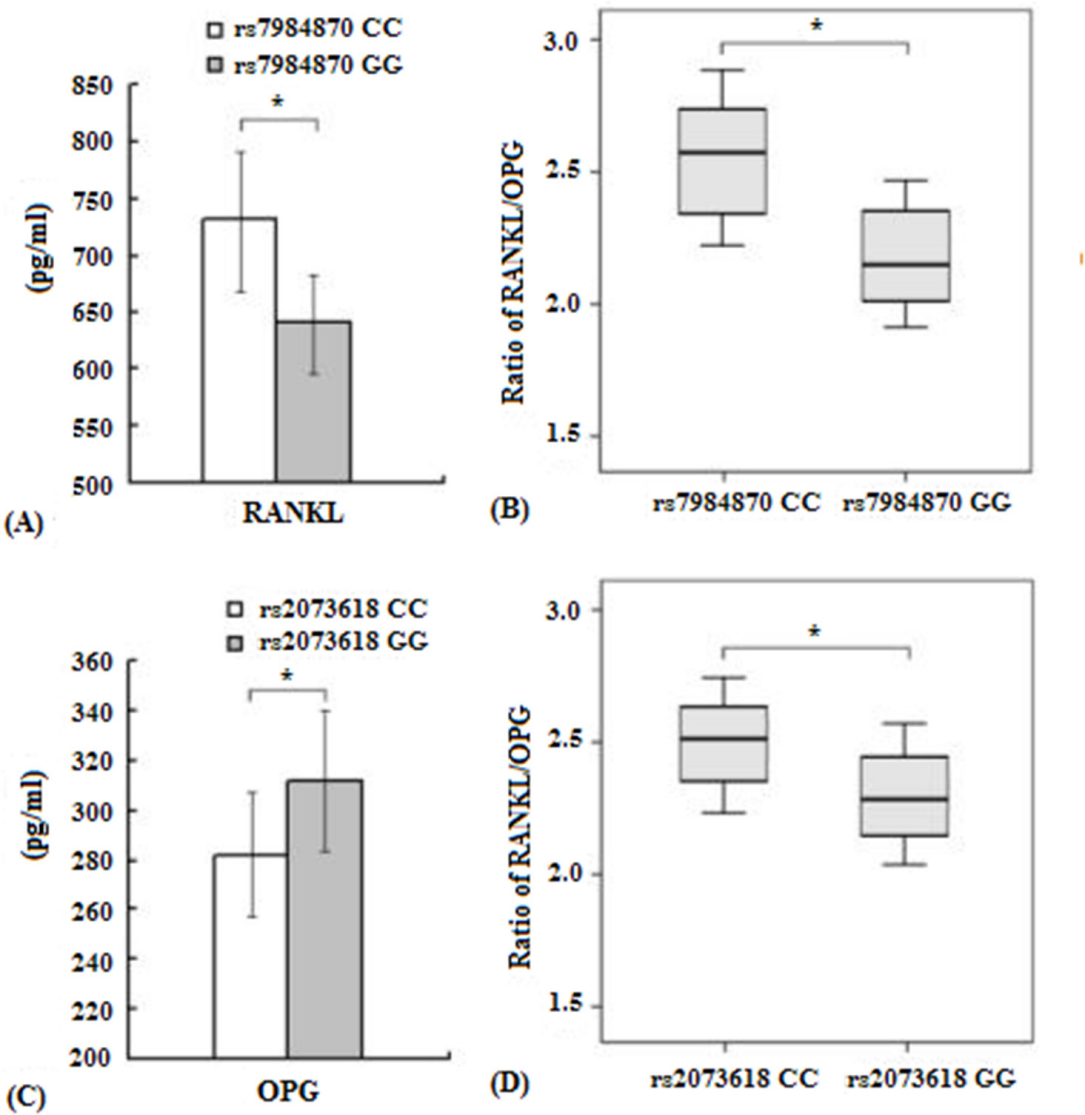
Association of RANKL and OPG genotypes with their serum levels and RANKL/OPG ratio. A, B. Soluble RANKL levels and the ratio of RANKL/OPG in serum samples of breast cancer patients with AI-related MS-AEs with either the CC genotype or GG genotype of the RANKL promoter SNP rs7984870 (CC = 76 vs. GG = 32). C, D. Soluble OPG levels and the ratio of RANKL/OPG in serum samples of breast cancer patients with AI-related MS-AEs with either the CC genotype or GG genotype of the OPG SNP rs2073618 (CC = 46 vs. GG = 59). *p < 0.05.

Using ELISA, we sought to investigate whether plasma levels of soluble RANKL and the ratio of RANKL/OPG were associated with the rs7984870 genotype in breast cancer patients with AI-related MS-AEs. Interestingly, we found that soluble RANKL levels were higher in breast cancer patients with AI-related MS-AEs carrying the rs7984870 CC genotype (739.3 ± 66.6 pg/ml) than those carrying the GG genotype (642.2 ± 57.8 pg/ml, p < 0.05, Fig 2A). Additionally, carriers of the rs7984870 CC genotype were more likely to have a higher RANKL/OPG ratio (2.58 ± 0.21) than carriers of the GG genotype (2.14 ± 0.12, p < 0.05, Fig 2B).

### The rs2073618 SNP may affect OPG expression and the ratio of RANKL/OPG

We subsequently tested whether the rs2073618 genotypes were correlated with OPG expression and the ratio of RANKL/OPG. Compared with the rs2073618 GG genotype (316.3 ± 43.7 pg/ml), carriers of the rs2073618 CC genotype had significantly lower OPG expression (282.2 ± 26.9 pg/ml, Fig 2C). Furthermore, carriers of the rs2073618 CC genotype were more likely to have a higher RANKL/OPG ratio (2.52 ± 0.18) than those with the GG genotype (2.30 ± 0.15, p < 0.05, Fig 2D).

### Lumbar spine BMD measurements and its correlation to the ratio of RANKL/OPG

**Fig 3:**
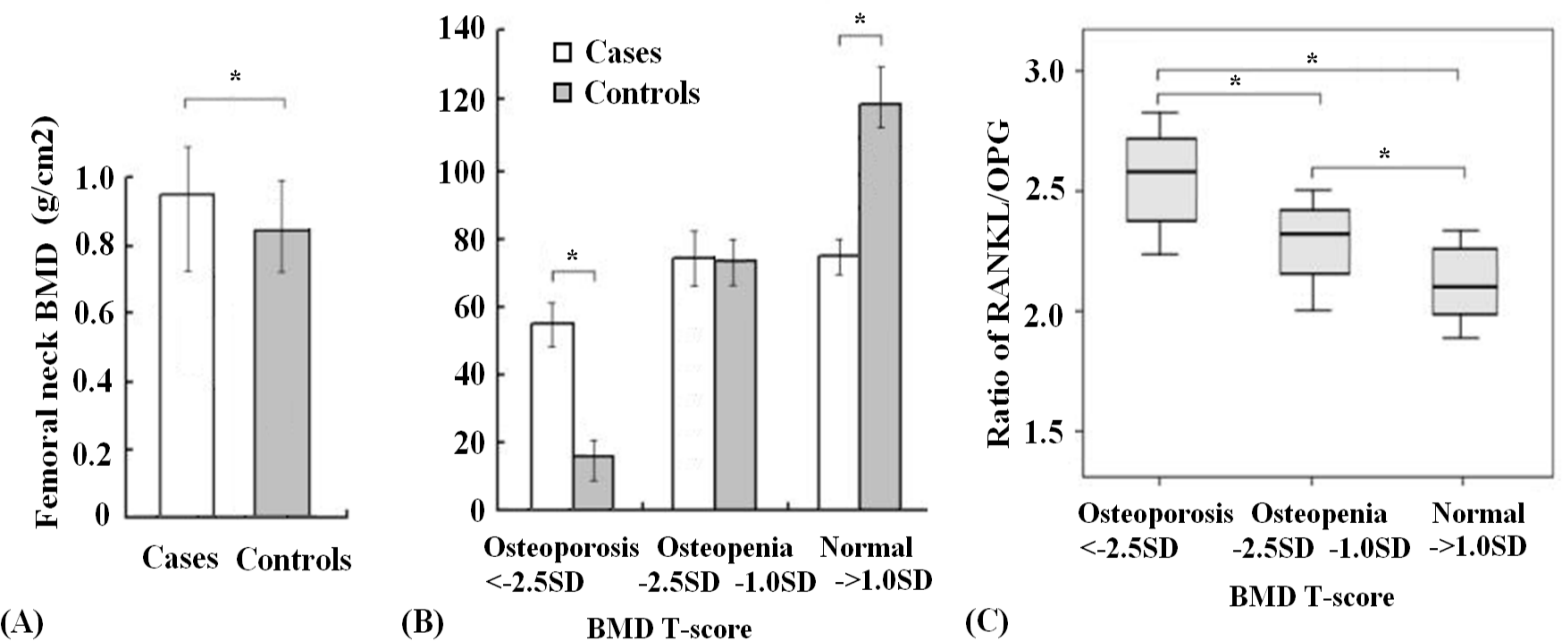
Lumbar spine BMD measurements and their correlations with the RANKL/OPG ratio in patients. A. Lumbar spine BMD absolute measurements in cases and controls. B. The prevalence of osteoporosis, osteopenia and normal BMD in patients with or without AI-related MS-AEs based on the BMD T score. C. Association of the lumbar spine BMD T score with the ratio of serum RANKL/OPG in the AI-related MS-AEs group.

Fig 3A shows the absolute measurements of the lumbar spine BMD of the study population. Compared with the control group, a significant decrease in the BMD in the AI-related MS-AEs group was observed (0.958 ± 0.273 g/cm^2^ vs. 0.853 ± 0.221 g/cm^2^, p < 0.05). The rates of osteoporosis, osteopenia and normal BMD in patients with or without AI-related MS-AEs based on the lumbar spine BMD T score are summarized in Fig 3B. Patients in the AI-related MS-AEs group had a higher prevalence of osteoporosis than the controls (27.4% vs. 8.5%, respectively, p < 0.05). In contrast, a normal BMD was more common in the control group. The prevalence of osteopenia was 37.0% in the AI-related MS-AEs group and 35.4% in the control group; however, this difference was not significant.

Furthermore, we investigated whether the lumbar spine BMD T score was correlated with the ratio of serum RANKL/OPG in the AI-related MS-AEs group (Fig 3C). As expected, we found that patients with osteopenia were far more likely to have a higher RANKL/OPG ratio than women with a normal BMD (2.35 ± 0.18 vs. 2.12 ± 0.15, respectively, p < 0.05). However, patients with osteoporosis were more likely to have a higher RANKL/OPG ratio compared with those with osteopenia (2.56 ± 0.25 vs. 2.35 ± 0.18, respectively, p < 0.05).

## Discussion

Aromatase inhibitor therapy in postmenopausal women causes bone health problems, such as lower BMD, vitamin D insufficiency, arthralgia and fractures. AI-induced bone health problems are increasingly recognized as a clinically significant toxicity of AI therapy, but the mechanisms underlying the development of these problems remain unclear. One study found that denosumab was an effective agent for the management of AI-induced bone loss, with an adverse event profile similar to that of placebo [18]. Loss of bone mass resulting from treatment with aromatase inhibitors is not unusual. However, osteoporosis in such patients is typically diagnosed too late, usually after a fracture appears. Arthralgia, muscle pain, bone pain and other musculoskeletal problems are typically the early symptoms of AI-induced skeletal damage. Fortunately, most skeletal damage is reversible with early supplementation of vitamin D and calcium. Therefore, to evaluate methods for identifying high-risk individuals and improving AI-induced bone health, we selected patients who experienced at least one of the following six symptoms of MS-AEs: joint pain, muscle pain, bone pain, arthritis, diminished joint function, or other musculoskeletal problems.

The ESCEO working group recommends treatment with denosumab and zoledronic acid to prevent bone loss and fractures in postmenopausal women treated with aromatase inhibitors for breast cancer [19]. Denosumab selectively inhibits RANKL, a primary mediator of the formation, resorptive function, and survival of osteoclasts. RANK/RANKL interactions lead to both differentiation and activation of osteoclasts, and the interaction of OPG with RANKL negatively regulates this process. In this study, we observed significant differences in the serum concentration of RANKL and the ratio of RANKL/OPG during AI therapy between cases and controls, suggesting that AI-related MS-AEs are associated with the RANKL/RANK/OPG bone remodeling pathway. Moreover, concentrations of the bone turnover markers CTX and PINP were elevated in the AI-related MS-AEs group compared with the control group. These results suggest that individuals with AI-related MS-AEs have a predisposition for higher bone resorption. The regulation of bone mass is governed by a complex interplay between bone-forming cells termed osteoblasts and bone-resorbing cells termed osteoclasts [20]. Osteoclast activation, which can lead to bone pain, fracture and other musculoskeletal problems, is induced by RANKL and suppressed by OPG.

Multiple factors influence the levels of RANKL and OPG. Smoking is one factor that affects the expression of RANKL and OPG; patients who are cigarette smokers tend to have lower serum concentrations of RANKL and OPG than non-smoking patients [21]. In our study, no significant difference was observed between the case group and control group based on smoking status. In addition to smoking, hormones can also influence the levels of RANKL and OPG; estrogen (and androgen) increases OPG secretion from osteoblasts, decreases the ratio of RANKL to OPG, downregulates the expression of RANKL on osteoclasts, and inhibits overall bone resorption [22,23]. Conversely, when estrogen and androgen levels decrease either as a consequence of normal menopause and aging or due to the effects of cancer treatments (AIs, chemotherapy, gonadotropin-releasing agonists, and androgen-deprivation therapy [ADT]), RANKL levels increase, and OPG levels decrease, which causes net bone resorption. In this study, a significant decrease was observed in mean estradiol levels in the AI-related MS-AEs group compared with the control group. Estradiol concentrations in postmenopausal women with early stage, ER-positive breast cancer who received anastrozole therapy significantly decreased over 24 months [24]. In contrast, levels of the serum bone turnover markers CTX and PINP increased significantly in the first 12 months of anastrozole treatment. One possible explanation for AI-related MS-AEs could be the presence of secondary bone disorders caused by decreases in the serum estradiol levels. However, not every patient treated with AIs develops these side effects, suggesting that some individuals may be predisposed to the negative skeletal effects of AIs [25]. Many studies have identified genetic variants in the RANKL, RANK, and OPG genes that are associated with bone mineral density and the risk for bone fractures [16,26]. Therefore, we hypothesized that SNPs related to markers of bone and joint health may contribute to AI-related MS-AEs.

We examined 29 SNPs using the “Tagger” program and data from the international HapMap project to investigate the relationship between RANKL/RANK/OPG polymorphisms and AI-related MS-AEs. Circulating T cells in the blood display detectable polymorphisms, and sufficient frequency differences in the alleles can be detected. Blood samples are easier to collect than tumor tissues and bone marrow; therefore, we detected the RANKL, RANK and OPG polymorphisms in easily accessible peripheral blood samples. We found that the SNPs rs7984870 of RANKL and rs2073618 of OPG were significantly associated with AI-related MS-AEs; however, we failed to find any significant association between any phenotype and RANK polymorphisms. Neither of these two SNPs had been tested previously in association with AI-related MS-AEs, but they had been associated previously with other bone diseases, such as rheumatoid arthritis (RA), osteonecrosis and fractures. Previous studies have reported that the rs7984870 SNP was consistently associated with an earlier age of RA onset in 3 independent seropositive (RF or anti-cyclic citrullinated peptide antibody positive) RA cohorts but not in seronegative RA patients [27]. Some of the OPG SNPs (rs10505348, rs2073617 and rs2073618) have previously been found to correlate with lumbar spine BMD in a large GWAS meta-analysis, with significance at the genome-wide level [28,29]. In addition, another study of the OPG rs2073618 SNP (1181 G/C) describes a 26% higher risk of hip fractures and a 52% higher chance of femoral neck fractures in women with the CC homozygote than women with the GG homozygote, independent of BMD [17].

In this study, we found that women with the rs7984870 CC or rs2073618 CC genotypes were at risk for AI-related MS-AEs. They also experienced a greater increase in the levels of CTX and PINP, which suggests enhanced bone resorption. In addition, the rs2073618 CC risk genotype but not the rs7984870 CC risk genotype was associated with a significant reduction in BMD at the lumbar spine. Similarly, women with any risk genotypes (rs2073618 CC or rs7984870 CC) of AI-related MS-AEs had a higher prevalence of osteoporosis and osteopenia than women with no risk genotypes. Similar results have been reported in another study; the minor allele of rs2073618 (C) was associated with higher levels of both PINP and CTX-I and a lower lumbar spine BMD [28]. Compared with the controls, a significant decrease in the mean estradiol levels was found in the AI-related MS-AEs group, but no significant differences in estradiol levels were observed between women with the risk genotypes or no risk genotypes. The profound estrogen deficiency induced by AIs is believed to account for the increased bone loss, fractures and incidence of musculoskeletal pains. Our findings in the current study confirmed that women with the risk genotypes could be more sensitive to hormonal manipulation than women with the no risk genotypes.

The association between serum levels of OPG and soluble RANKL and their gene polymorphisms has been investigated in several studies [30–32]. The risk C allele of rs7984870 has been reported to confer a 2-fold higher level of plasma soluble RANKL in RF+ RA patients and significantly elevated mRNA expression of RANKL isoform1 by activated control T cells. Furthermore, this study suggested that the minor C allele of rs7984870 creates a binding site for the transcription factor SOX5, which may influence bone metabolism and RANKL transcription [27]. Our study had a similar outcome; the risk CC genotype of rs7984870 conferred a higher plasma soluble RANKL level and RANKL/OPG ratio. In addition, a study of a Chinese population showed that the Asn-Asn genotype of rs2073618 is associated with lower serum OPG levels compared with the Lys-Lys genotype [30], which is consistent with our finding that the C allele of rs2073618 is associated with lower serum OPG levels and a higher RANKL/OPG ratio. Moreover, a significant correlation was observed between the ratio of RANKL/OPG and BMD in the AI-related MS-AEs group. OPG acts as a decoy receptor and blocks the effects of RANKL. RANKL increases the production, activity, and survival of osteoclasts [12]. Therefore, SNPs that lead to the decreased activity of OPG would be expected to have a negative impact on bone health due to increased bone resorption.

Following one year of aromatase inhibitor therapy, a 56-year-old breast cancer patient presented with widespread osteoarthralgia, and serum analysis showed increased expression of RANKL and an altered distribution of Th1/Th2 lymphocytes [33]. Another study indicated that IL17RA may also increase in patients with severe musculoskeletal symptoms after therapy with aromatase inhibitors [34]. Considering the role of estrogen in the immune system, we also tested the expression of some pro- and anti-inflammatory markers in our study. In contrast to our original hypothesis, we observed no significant differences in the serum concentrations of inflammatory markers during AI therapy between cases and controls. This result is similar to the findings of another study [35]. However, we cannot exclude the possibility that AI-related MS-AEs are due to a systemic inflammatory response. Other studies have suggested that local inflammatory processes, such as tenosynovitis at the wrist, may be occurring, as demonstrated using magnetic resonance imaging, musculoskeletal sonography and electromyography [36,37]. Therefore, future studies should investigate the local concentration of inflammatory factors.

To summarize, these findings add to our knowledge of the role of genetic variation in the RANKL/RANK/OPG signaling pathway in AI-related MS-AEs. Increased levels of bone turnover markers and a higher RANKL/OPG ratio suggest disturbances between bone formation and resorption in patients with AI-related MS-AEs. Patients with the CC genotype of the RANKL rs7984870 SNP appeared to be at risk of having higher serum RANKL levels and a higher RANKL/OPG ratio, whereas the CC genotype of the OPG rs2073618 SNP was associated with lower serum OPG levels and a higher RANKL/OPG ratio. In both cases, a higher RANKL/OPG ratio may negatively impact bone health due to increased bone resorption. Furthermore, these two risk genetic variations in RANKL and OPG also affected bone turnover and BMD. We could not compare our data with other studies of AI-related MS-AEs because these two SNPs have not been investigated previously. Therefore, it will be important to carry out studies in larger cohorts of Chinese postmenopausal women and in different populations worldwide to confirm our results.

## Supporting Information

S1 Fig Linkage disequilibrium between genotyped SNPs of RANK (A), OPG (B) and RANKL (C). (TIF)

S1 Table Serum estradiol, CTX, PINP, OPG, RANKL concentration and BMD prior aromatase inhibitors treatments. (DOC)
