# Supplementary figures and images for: RANKL and OPG Polymorphisms Are Associated with Aromatase Inhibitor-Related Musculoskeletal Adverse Events in Chinese Han Breast Cancer Patients
Source: PLoS One. 2015 Jul 28;10(7):e0133964. doi: 10.1371/journal.pone.0133964 (PMC4547828; doi:10.1371/journal.pone.0133964)

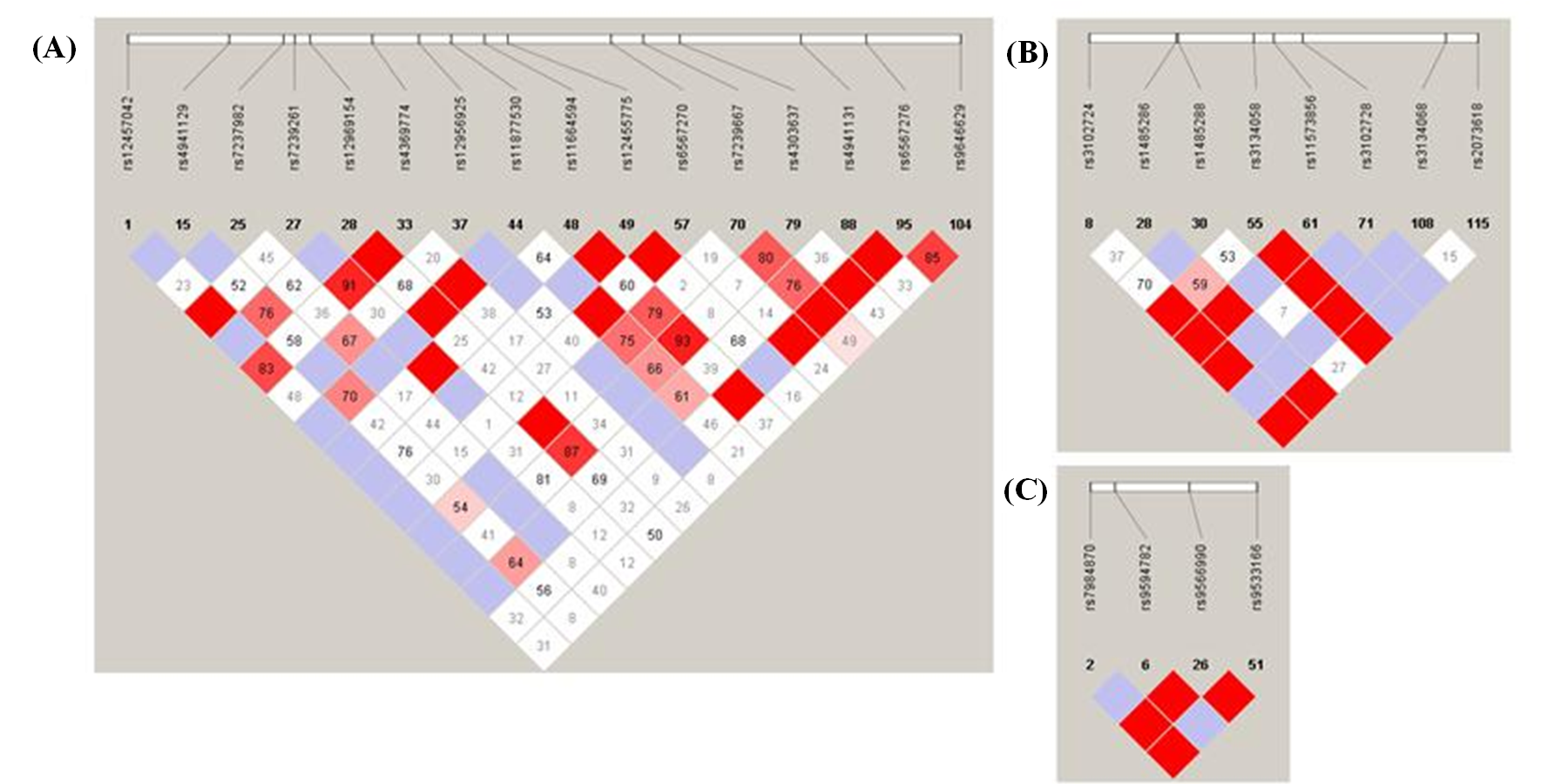

Supplement: S1 Fig — (TIF) [file pone.0133964.s001.tif]
