# Supplementary material for: RANKL and OPG Polymorphisms Are Associated with Aromatase Inhibitor-Related Musculoskeletal Adverse Events in Chinese Han Breast Cancer Patients
Source: PLoS One. 2015 Jul 28;10(7):e0133964. doi: 10.1371/journal.pone.0133964 (PMC4547828; doi:10.1371/journal.pone.0133964)
Supplement: S1 Table — (DOC) [file pone.0133964.s002.doc]

**Table S1** **Serum estradiol, CTX, PINP, OPG, RANKL concentration and BMD prior aromatase inhibitors treatments**

**Cases Controls**

**(mean ± SD) (mean ± SD) p value**

**E2 (pg/ml)** 16.51 ± 8.26 17.12 ± 8.08 0.492

**CTX (pg/ml)** 202.7 ± 107.5 198.6 ±101.6 0.525

**PINP (pg/ml)** 312.3 ± 121.8 307.2 ± 113.7 0.486

**OPG (pg/ml)** 338.6 ± 127.5 345.2 ±119.8 0.253

**RANKL (pg/ml)** 429.0 ± 132.2 417.9 ± 119.9 0.281

**Lumbar spine BMD (g/cm2)**  1.051 ± 0.257 1.065 ± 0.242 0.225

Note: SD= standard deviation; E2= estradiol.
